# Supplementary material for: Antibiotic treatment failure in children aged 1 to 59 months with World Health Organization-defined severe pneumonia in Malawi: A CPAP IMPACT trial secondary analysis
Source: PLoS One. 2022 Dec 14;17(12):e0278938. doi: 10.1371/journal.pone.0278938 (PMC9750006; doi:10.1371/journal.pone.0278938)
Supplement: Attachment — Submitted filename: Response to Reviewers.pdf [file pone.0278938.s006.pdf]

6 October 2022

To: The Editor-in-Chief,

PLOS ONE

Resubmission: Manuscript Submission: [PONE-D-22-10313]

We would like to thank you for reviewing our manuscript Ref: [PONE-D-22-10313] - [EMID:cf85d3bc2844e620].

With reference to feedback provided on 3 August 2022, we have made edits to the manuscript in response to the comments to conform the manuscript to the PLOS ONE journal. We have also made edits in relation to comments made by the 3 reviewers.

(A) Responses to editors (responses in italic font):

1. Please ensure that your manuscript meets PLOS ONE's style requirements, including those for file naming. The PLOS ONE style templates can be found at https://journals.plos.org/plosone/s/file?id=wjVg/PLOSOne_formatting_sample_main_body.pdf and https://journals.plos.org/plosone/s/file?id=ba62/PLOSOne_formatting_sample_title_authors_affiliations.pdf

> *The manuscript has been updated accordingly (this includes inserting initials next to the contact details of the corresponding author on page 1 and adjusting font size for level 1 and level 2 subheadings).*

2. Please provide additional details regarding participant consent. In the ethics statement in the Methods and online submission information, please ensure that you have specified what type you obtained (for instance, written or verbal, and if verbal, how it was documented and witnessed). If your study included minors, state whether you obtained consent from parents or guardians. If the need for consent was waived by the ethics committee, please include this information.

> *This has been updated in the methods section of the manuscript (lines 222-224 of the clean version and lines 226-228 of the track changes version).*

3. Thank you for stating the following in the Acknowledgments Section of your manuscript:

The Bill & Melinda Gates Foundation (OPP1123419), International AIDS Society (141022) and Health Empowering Humanity provided funding for CPAP IMPACT.

However, funding information should not appear in the Acknowledgments section or other areas of your manuscript. We will only publish funding information present in the Funding Statement section of the online submission form.

Please remove any funding-related text from the manuscript and let us know how you would like to update your Funding Statement. Currently, your Funding Statement reads as follows:

EDM: The Bill & Melinda Gates Foundation (OPP1123419) (https://www.gatesfoundation.org/) and a CIPHER grant from the International AIDS Society (141022) (https://www.iasociety.org/HIV-Programmes/Programmes/Paediatrics-CIPHER/CIPHER-Grant-Programme.)

The sponsor had no role in study design, data collection and analysis, decision to publish, or preparation of the manuscript.

Please include your amended statements within your cover letter; we will change the online submission form on your behalf.

> *Thank you for your guidance on this. The text on the funding information has been deleted from the manuscript. Please insert the following text onto the online submission page:*
>
> *"EDM: The Bill & Melinda Gates Foundation (OPP1123419) (https://www.gatesfoundation.org/) and a CIPHER grant from the International AIDS Society (141022) (https://www.iasociety.org/HIV-Programmes/Programmes/Paediatrics-CIPHER/CIPHER-Grant-Programme.)*
>
> *The sponsor had no role in study design, data collection and analysis, decision to publish, or preparation of the manuscript."*

4. We note that you have stated that you will provide repository information for your data at acceptance. Should your manuscript be accepted for publication, we will hold it until you provide the relevant accession numbers or DOIs necessary to access your data. If you wish to make changes to your Data Availability statement, please describe these changes in your cover letter and we will update your Data Availability statement to reflect the information you provide.

> *On this re-submission we would like to request to state that all data used for this analysis is available and has been submitted as supplemental files S1 (Data), S2 (Data dictionary), S3(Codebook) and S4 (Analysis instructions do file for reproducing analysis results). The manuscript has been updated to indicate these supplemental files submitted (lines 463-467 of the clean version and lines 587-591 of the track changes version).*

5. Please include a separate caption for figure 1 in your manuscript.

> This has now been added *(line 254 of the clean version and lines 266 of the track changes version).*

(B) Responses to reviers (responses in italic font):

Reviewer #1: The authors conducted a secondary analysis to investigate risk factors for antibiotic failure among children hospitalized with pneumonia. They analyzed 538 children and identified higher odds of treatment failure in children with SAM, pre-hospital antibiotic use, and with general danger sign of hospitalization.

1. Abstract. "Children in this analysis were alive and not receiving ceftriaxone by hospital day 2." Why exclude these children? Will it be more appropriate to include them as treatment failure?

As stated under the "study population and procedures" sub-heading section of the methods section, benzylpenicillin and gentamicin were the first line antibiotic regimen for pneumonia. Ceftriaxone was the second line antibiotic regimen for pneumonia. The aim was to analyze treatment failure of the pneumonia antibiotic regimen that is indicated to be commenced on hospitalization.

Some patients with features of possible meningoencephalitis were commenced on high dose ceftriaxone for possible meningitis (as specified in lines 155-158 of the clean version manuscript and lines 165-168 of track changes version manuscript). These were excluded from analysis since we primarily aimed at looking at treatment failure to the first line pneumonia regimen for hospitalized children. We have now added more detail on additional criteria for exclusion from analysis (death before day 2, withdrawal from the trial), both in the abstract and the methods section.

2. Line 217. "the adjusted adds ratios" has typo?

This typo error has been corrected.

3. Line 224 "p values between 0.05 and 0.10 indicated a weak trend to association". Please avoid to use "trend to association" as there might cause confusion about trend test.

We have revised this sentence (lines 242-243 of the clean version manuscript and lines 254-255 of track changes version manuscript).

4. Table 1. The proportion reported within the study arm may not be appropriate. It would be more appropriate to report the proportion of no treatment failure within bCPAP and so on rather reporting the proportion of bCOAP within no treatment failure group.

Table 1 has been revised to address this.

5. Table 1. The concern raised in point 4 applies to many other variables. Besides, if follow the logic of the presentation, how come the proportions don't add up to 1 for some characteristics, 3 doses of hib and so on.

Table 1 has been revised to address this.

6. Line 273. "each increase of hemoglobin ….decreased the odds ratio…" Hemoglobin is not an intervention so it's questionable to imply casual effect here. Similar concern applies to other predictors and other texts as well.

The results section has been revised in relation to this when stating odds ratios.

Reviewer #2: This paper is very important and is worthy publishing for wider audience especially due to the etiological and epidemiological shifts of Pneumonia cases characteristics due to many interventions done so far in Malawi and beyond.
I have a very few comments which the authors may consider them;
1. Sentence on line number 104-105 is not clear. Please check it

This second paragraph of the introduction has been edited.

2. I think this paper can best benefit from the recent published on Malawian data (https://www.ncbi.nlm.nih.gov/pmc/articles/PMC8323352/) for instance on introduction paragraph 3 there is no clear supporting evidence of the arguments but this paper can help.

Thank you for bringing this article to our attention. This has now been referred to in the manuscript *(lines 127 of the clean version manuscript and lines 133-137 of track changes version manuscript).*

3. In your finding, there is and indication that prior antibiotic intake is a predictor of antibiotic treatment failure, did you capture the details of the type of antibiotic taken?? If yes please provide the details if not the need to include this on the limitation as there is no scientific link or explanation on this finding.

Thank you for raising this question. Unfortunately, we did not capture details of the prior antibiotic regimen. We have now included this as a limitation (lines 445-446 of the clean version manuscript and lines 555-558 of track changes version manuscript).

4. I feel your discussion is unnecessarily long due to inclusion of some aspects which are not significant finding in your study. You may consider focusing on the key and significant findings

We have edited our discussion remarkably to address this. Edits have included

- *Deleting a paragraph that previously discussed hypoxemia and general danger signs*
- *Deleting discussion points on anemia*
- *Deleting discussion text on early versus late treatment failure*
- *Minimizing text on general danger signs in relation to treatment failure*
- *Bringing more focus of the analysis and conclusion towards severe acute malnutrition and prior antibiotic use*

Reviewer #3: Comments
Thank you for invitation to review Antibiotic treatment failure in children aged 1 to 59 months with World Health Organization-defined severe pneumonia in Malawi: a CPAP IMPACT trial secondary analysis. The article is presented in a structured way and is well written. The research has ethical approval and meets the applicable standards for ethics and research integrity. Generally, the paper is well written and structured however in my opinion the paper has shortcomings in regards the abstract, introduction, and methods parts. Below I provide some remarks on the introduction and method parts. Additionally, I suggest the authors to discuss the implication of the result.

1. In the abstract section, avoid use of abbreviations (CPAP, SAM, and OR). Similarly in abstract section, methods are not explanatory, try to indicate method of data analysis.

We have deleted most of the abbreviations from the abstract. Minimal abbreviations (e.g., aOR) now appear.

2. In the abstract section, your conclusion should be based on your findings.

The abstract conclusion has been edited to address this by directly relating treatment failure severe acute malnutrition on possible questions diagnosis etiology.

3. In the introduction section, you need to show the previous works, how your finding adds to the existing knowledge, and gap of your work. Furthermore, you need to show the impact of the problem using figures rather than words. Generally, your introduction section lacks figures.

We have edited the introduction by adding more figures and proportions to reflect impact of the problem, previous work and previously reported risk factors.

4. On the method part, it is too shallow and it didn't address all important components of research methodology. You didn't mention the study period. You need to explain in detail about the study area. How you control possible confounders? You didn't mention quality control measures. You need to show how you measure your outcome variable in detail.

We have now edited the methods section as follows

- *Addition of detail of the study period (line 146 of the clean version manuscript and line 156 of track changes version manuscript)*
- *Addition of detail of the study area (lines 138-141 of the clean version manuscript and lines 148-151 of track changes version manuscript),*
- *Some text describing quality control measures have been added (lines 186-192 of the clean version manuscript and lines 199-205 of track changes version manuscript)*
- *The data analysis sub-section of the methods section has been edited to reflect analytical methods used.*

5. On the result part, I recommend the author to write the outcome variable in detail rather than discussing supporting evidences. I suggest the authors to limit the usage of abbreviations in result section.

The results section has been edited. The only abbreviations appearing now are those already appearing in sections prior.

6. The discussion is well written and structured. However, it lacks the implication of the results.

We have now expanded the conclusion section (by relating our findings to implications now reflecting that complex issues like malnutrition need to be addressed to improve pneumonia outcomes).

Yours sincerely,

Tisungane Mvalo

Address for correspondence: Tisungane Mvalo, MBBS, MMED, FC Paed. UNC Project Malawi, Private Bag A104, Kamuzu Central Hospital, Lilongwe, Malawi.
Phone: +265 9 99 917 355.Fax: +2651755954.Email: tmvalo@unclilongwe.org
