# Supplementary material for: Antibiotic treatment failure in children aged 1 to 59 months with World Health Organization-defined severe pneumonia in Malawi: A CPAP IMPACT trial secondary analysis
Source: PLoS One. 2022 Dec 14;17(12):e0278938. doi: 10.1371/journal.pone.0278938 (PMC9750006; doi:10.1371/journal.pone.0278938)
Supplement: Attachment — Submitted filename: comment for Plos One.docx [file pone.0278938.s005.docx]

**Comments**

Thank you for invitation to review Antibiotic treatment failure in children aged 1 to 59 months with World Health Organization-defined severe pneumonia in Malawi: a CPAP IMPACT trial secondary analysis. The article is presented in a structured way and is well written. The research has ethical approval and meets the applicable standards for ethics and research integrity. Generally, the paper is well written and structured however in my opinion the paper has shortcomings in regards the abstract, introduction, and methods parts. Below I provide some remarks on the introduction and method parts. Additionally, I suggest the authors to discuss the implication of the result.

1. In the abstract section, avoid use of abbreviations (CPAP, SAM, and OR). Similarly in abstract section, methods are not explanatory, try to indicate method of data analysis.
2. In the abstract section, your conclusion should be based on your findings.
3. In the introduction section, you need to show the previous works, how your finding adds to the existing knowledge, and gap of your work. Furthermore, you need to show the impact of the problem using figures rather than words. Generally, your introduction section lacks figures.
4. On the method part, it is too shallow and it didn’t address all important components of research methodology. You didn’t mention the study period. You need to explain in detail about the study area. How you control possible confounders? You didn’t mention quality control measures. You need to show how you measure your outcome variable in detail.
5. On the result part, I recommend the author to write the outcome variable in detail rather than discussing supporting evidences. I suggest the authors to limit the usage of abbreviations in result section.
6. The discussion is well written and structured. However, it lacks the implication of the results.
